# Genomic basis of schistosome resistance in a molluscan vector of human schistosomiasis

**DOI:** 10.1016/j.isci.2024.111520

**Published:** 2024-12-02

**Authors:** Si-Ming Zhang, Guiyun Yan, Abdelmalek Lekired, Daibin Zhong

**Affiliations:** 1 Center for Evolutionary and Theoretical Immunology, Department of Biology, University of New Mexico, Albuquerque, NM 87131, USA; 2 Program in Public Health, College of Health Sciences, University of California, Irvine, Irvine, CA 92697, USA

**Keywords:** Disease, Molecular biology, Parasitology

## Abstract

Freshwater snails are obligate intermediate hosts for the transmission of schistosomiasis, one of the world’s most devastating parasitic diseases. To decipher the mechanisms underlying snail resistance to schistosomes, recombinant inbred lines (RILs) were developed from two well-defined homozygous lines (iM line and iBS90) of the snail *Biomphalaria glabrata*. Whole-genome sequencing (WGS) was used to scan the genomes of 46 individual RIL snails, representing 46 RILs, half of which were resistant or susceptible to *Schistosoma mansoni*. Genome-wide association study (GWAS) and bin marker-assisted quantitative trait loci (QTLs) analysis, aided by our chromosome-level assembled genome, were conducted. A small genomic region (∼3 Mb) on chromosome 5 was identified as being associated with schistosome resistance, designated the *B. glabrata* schistosome resistance region 1 (BgSRR1). This study, built on our recently developed genetic and genomic resources, provides valuable insights into anti-schistosome mechanisms and the future development of snail-targeted biocontrol programs for schistosomiasis.

## Highlights

• Recombinant inbred (RI) lines (RILs) of *Biomphalaria glabrata* were created
• Forty-six RIL snails, each representing a different RIL, were sequenced individually
• GWAS and bin marker-assisted QTL analyses were performed
• A 3 Mb schistosome-resistant region (BgSRR1), containing 118 genes, was identified

## Graphical abstract

**Figure:**
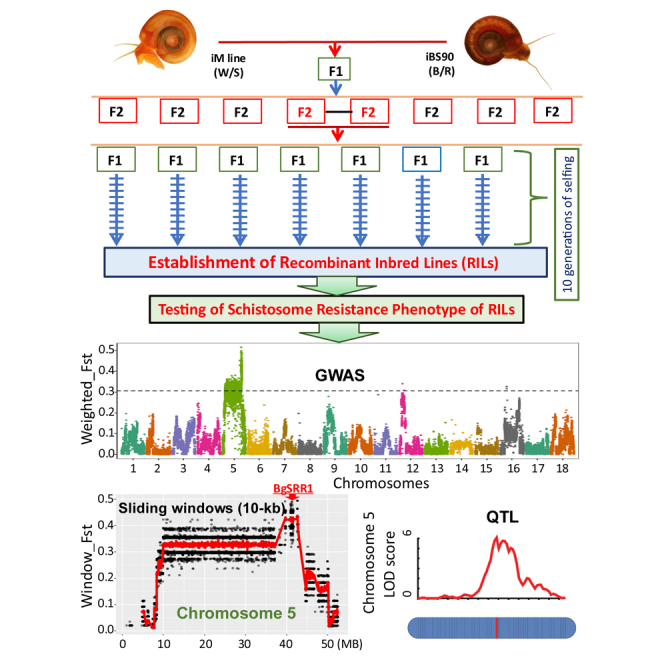

## Introduction

Schistosomiasis, one of the most devastating neglected tropical diseases, poses a persistent public health and economic challenge in the developing world.^1^^,^^2^^,^^3^ It has recently spread from developing countries to European nations due to human migration and climate change.^4^^,^^5^ The disease causes significant illness and death,^6^ promotes the transmission of human immunodeficiency virus,^7^ and can lead to bladder cancer.^8^ Currently, there is no effective vaccine against schistosomes. The only available treatment is praziquantel (PZQ), a chemotherapy that has been in use for over 40 years.^9^ However, relying solely on PZQ-based control programs is unlikely to achieve disease control goals, as PZQ-treated patients, especially children, quickly become reinfected.^10^^,^^11^ Additionally, concerns about drug resistance in schistosomes, particularly in mass drug administration programs, are growing.^12^^,^^13^

Freshwater snails serve as obligate intermediate hosts for the digenetic trematodes *Schistosoma* spp., the causative agents of schistosomiasis. This is because the life cycle of schistosomes involves asexual and sexual developmental stages within a snail intermediate host and a mammalian definitive host, respectively. Snail control, alone or in combination with other strategies, has proven to be the most effective means of reducing schistosomiasis prevalence in endemic areas.^14^^,^^15^ However, the widely used molluscicide niclosamide has harmful effects on the aquatic ecosystem, as it is toxic to other aquatic animals.^16^^,^^17^ Given the critical role of snails in the aquatic ecosystem, an ideal biocontrol strategy should aim to disrupt parasite life cycles without eliminating the intermediate snail hosts. Field evidence supports this strategy, as the introduction of schistosome-resistant *Biomphalaria tenagophila* snails to endemic areas in Brazil has resulted in reduced disease transmission.^18^

*Biomphalaria glabrata* — *Schistosoma* *mansoni* has been used as a model system for studying the compatibility between snails and schistosomes, particularly snail resistance to schistosomes, since the mid-20th century.^19^ These studies have primarily focused on immunological responses, with significant progress made in recent omic-based research.^20^^,^^21^^,^^22^^,^^23^^,^^24^^,^^25^^,^^26^^,^^27^^,^^28^^,^^29^ It is well established that immunological responses have genetic bases.^30^^,^^31^ Previous studies have shown that snail resistance or susceptibility to schistosomes has a strong genetic component.^32^^,^^33^^,^^34^^,^^35^^,^^36^ Therefore, genetic mapping of schistosome resistance or susceptibility should offer valuable insights into these mechanisms and help elucidate the underlying immunological responses. This knowledge could potentially aid in developing biocontrol programs, which have shown promise in controlling vector-borne diseases through clustered regularly interspaced short palindromic repeats (CRISPR)-mediated gene drive technologies.^37^^,^^38^^,^^39^^,^^40^

A significant study on the genetic analysis of compatibility between snails and schistosomes using the *B. glabrata* — *S. mansoni* model was published by Charles Richards in 1970.^32^ Richards laid the foundation for understanding snail resistance to schistosomes through extensive classical crosses between snails with different resistance phenotypes. Genetic mapping using various mapping populations (most of which are pre-existing laboratory strains) and genotyping assays led to the identification of multiple resistant loci, located on different chromosomes,^41^^,^^42^^,^^43^^,^^44^^,^^45^^,^^46^ yielding valuable insights into the resistance mechanisms while also leaving unanswered questions (see details in the discussion section).

The success of genetic mapping relies heavily on the strategic design of mapping populations and genotyping technologies. Typically, classic mapping populations involve F2 offspring and backcrosses derived from crossing two inbred parents. Recombinant inbred (RI) lines or RILs, derived from the F2 population, offer distinct advantages for genetic mapping. RILs are created by crossing two parental strains with contrasting phenotypes followed by successive generations of inbreeding (selfing or full-sib mating). Meiotic crossover events result in a mosaic parental genome in each RI line, and subsequent inbreeding increases recombination events and leads to a rapid reduction in heterozygosity.^47^^,^^48^^,^^49^^,^^50^^,^^51^ RILs have commonly been used in plant genetics and breeding but have seldom been utilized in animal genetics, particularly for non-model organisms. This is mainly due to the labor-intensive, expensive, and time-consuming process of generating animal RILs, as well as the significant challenges associated with their maintenance.

We produced RILs from a cross between two well-defined homozygous lines of *B. glabrata*, the iM line and iBS90.^45^ To dissect this genetic resource, we employed whole-genome sequencing (WGS), a high-throughput genotyping assay, to scan every single nucleotide across the genomes of 46 individual RIL snails, representing 46 phenotyped RILs. This approach, designated as RIL-WGS, allowed us to reveal a significant number of single-nucleotide polymorphisms (SNPs) and bin markers for subsequent genome-wide association study (GWAS) and quantitative trait loci (QTLs) analysis. As a result, we identified a small genomic region and genes within that region involved in anti-schistosome defense.

## Results

### Development and phenotyping of RIL snails

**Figure 1:**
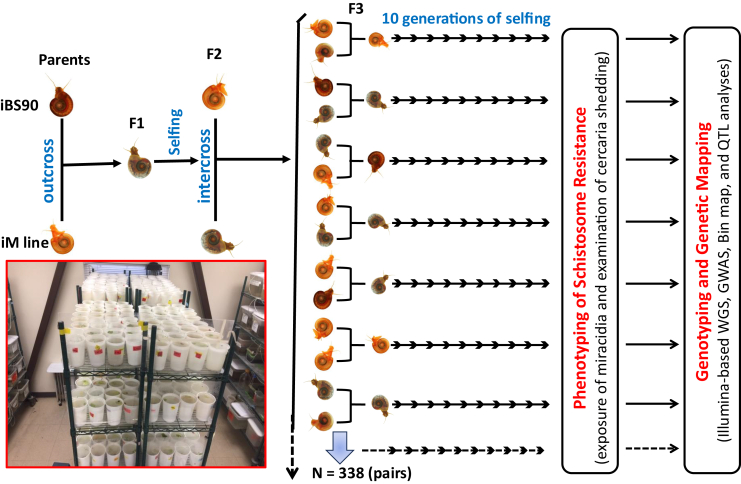
Breeding scheme of RIL snails and testing of their resistant or susceptible phenotype Homozygous iM line (albino and susceptible to schistosomes) and homozygous iBS90 (pigmented and resistant to schistosomes) snails were used as parent snails. Single iM line and iBS90 snails were placed in a 1 L plastic cup and allowed to produce F1 progeny. Since pigmentation is a dominant Mendelian trait, three possible outcomes were expected in F1 snails: albino F1 snails produced by selfing the iM line snail and pigmented F1 snails generated either from selfing the iBS90 snail or from a cross between the iM line and iBS90 snails. Albino F1 snails were discarded, while pigmented F1 snails were retained. These pigmented F1 snails were placed individually in plastic cups to produce F2 snails. To distinguish the two types of pigmented F1 snails (from selfing or crossing), we examined the colors of the F2 snails. If all F2 snails were pigmented, it suggested they originated from a single F1 snail through selfing of the parental iBS90, and they were discarded. If the F2 snails showed a mix of albino and pigmented individuals, it indicated that their parental F1 snail was produced from a cross between the iM line and iBS90 snails. The F2 snails were then retained for subsequent breeding. An albino F2 snail and a pigmented F2 snail from these F2 snails were randomly paired and placed in a plastic cup to produce offspring (an F1 population). From this F1 population, individual F1 snails were kept in plastic cups and allowed to self for 10 generations; in each generation, one snail was selected to produce the next generation through selfing. As a result, the RILs were obtained. Each RI line was tested for the resistance phenotype after miracidia exposure and cercarial shedding, as described in the STAR Methods section. All photographs presented in this figure and in the graphical abstract were prepared by S.-M.Z.

Two important biological characteristics of the *B. glabrata* – *S. mansoni* system contributed to the successful genetic design of the snail RILs. Firstly, the wild-type pigmentation follows Mendelian inheritance patterns, allowing us to confirm successful crosses. Secondly, *B. glabrata* is hermaphroditic, enabling both crossing and selfing. The breeding of snail RILs originated from an effort that spanned over 20 years at The University of New Mexico. During this period, the iM line and iBS90 were developed through 81 and 41 generations of selfing from a single M line and BS90 snail, respectively.^45^ Both lines were confirmed to be homozygous with contrasting resistance phenotypes and were used as parental snails to generate RILs. A total of 338 pairs, randomly generated from different F2 intercrosses, served as founders for subsequent selfing (Figure 1). Over more than 3.5 years, 137 RIL lines were obtained and tested for their phenotype. Among them, 118 RILs displayed a clear phenotype (42 resistant and 76 susceptible), while 19 had ambiguous phenotypes as only a portion of the snails in each line shed few cercariae. From the 118 RILs with clear phenotypes, we randomly selected 46 to represent 46 RI lines, with half (*n* = 23) being either resistant or susceptible to schistosomes.

### Quality control and evaluation of illumina reads

Since the susceptible iM line and the resistant iBS90 were used as the parental snails to generate the RIL population, the Illumina reads used to assemble the genomes of the two lines^45^ were retrieved for the current study. To ensure data comparability, the same quality control criteria described in the STAR Methods section were applied to all raw reads generated by 150 × 2 paired-end Illumina sequencing from the iM line, iBS90, and RILs. This resulted in 109.86, 118.34, and 664.4 Gb of clean reads from the iM line, iBS90, and RIL snails, respectively. Using our recently published chromosome-level assembly of *B. glabrata* as the reference genome,^52^ the mapping rates of the iM line, iBS90, and RIL snails to the reference genome were 99%, 97%, and 96%, respectively. The sequence coverages for the iM line, iBS90, and the RIL population were 125X, 132X, and 16X, respectively (Table S1).

### Identification of genome-wide SNPs

**Figure 2:**
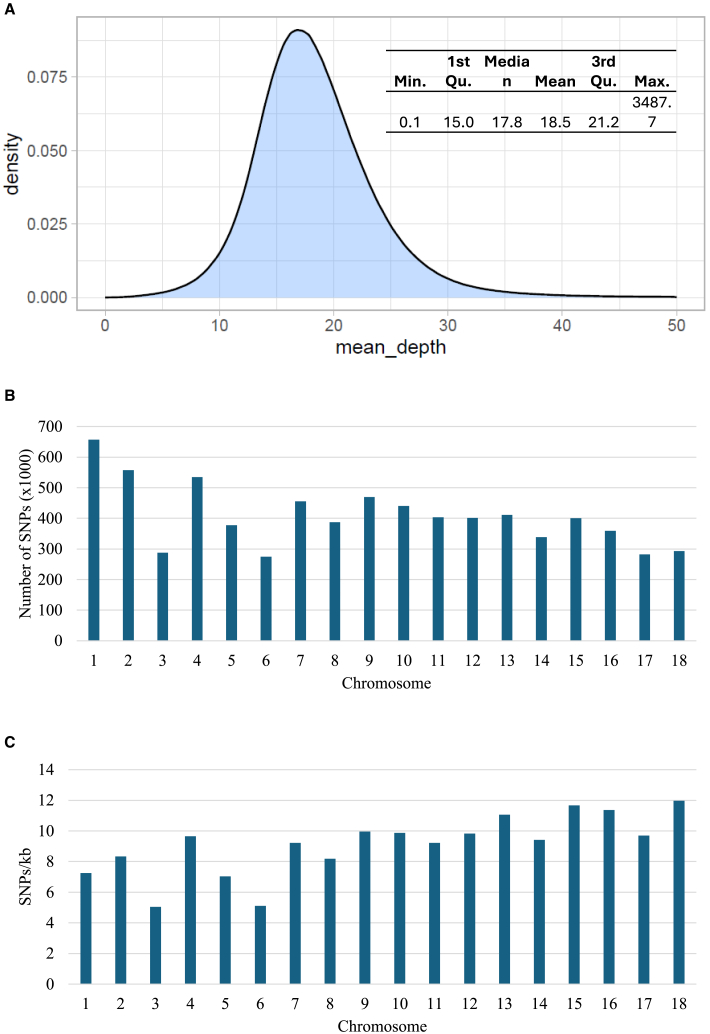
SNP analysis (A) Distribution of mean coverage (depth) for the 9,079,154 SNPs identified in the 46 RIL snails. (B) Number of SNPs across the 18 chromosomes. (C) Density of SNPs across the 18 chromosomes. Data are represented as mean ± SEM.

A total of 9,079,154 SNPs were identified between the iM line and iBS90 parental snails. Among these, 273,307 SNPs were found in coding regions (including upstream −5kb and downstream +2kb), with 119,241 SNPs resulting in nonsynonymous substitutions. These SNPs were used to genotype the RIL population using CLC genomics workbench. The resulting sequence variation data were exported as variant call format files, which were then combined using the Bcftools software package. The mean depth of SNP coverage for the iM line, iBS90, and RIL snails was 125X, 111X, and 14.20 ± 0.38X, respectively (Table S2). The distribution of SNP coverages is shown in Figure 2A. After filtering out markers with high missing genotype (>20%), low coverage (<5), and low minor allele frequency (<10%), a total of 7,330,259 SNP markers were retained for downstream analysis of the association between phenotype and genome-wide SNPs. The distribution of these high-quality SNPs across the 18 chromosomes shows a general correlation with genomic sizes (r = 0.71) (Figure 2B). The longest chromosome (chr 1) has the highest number of SNPs, while chromosome 6 has the lowest. The average SNP density across the 18 chromosomes is 9.11 ± 0.47/kb, with the highest density on chromosome 18 (11.97/kb) and the lowest on chromosome 3 (5.04/kb) (Figure 2C).

### GWAS and sliding-window analysis

**Figure 3:**
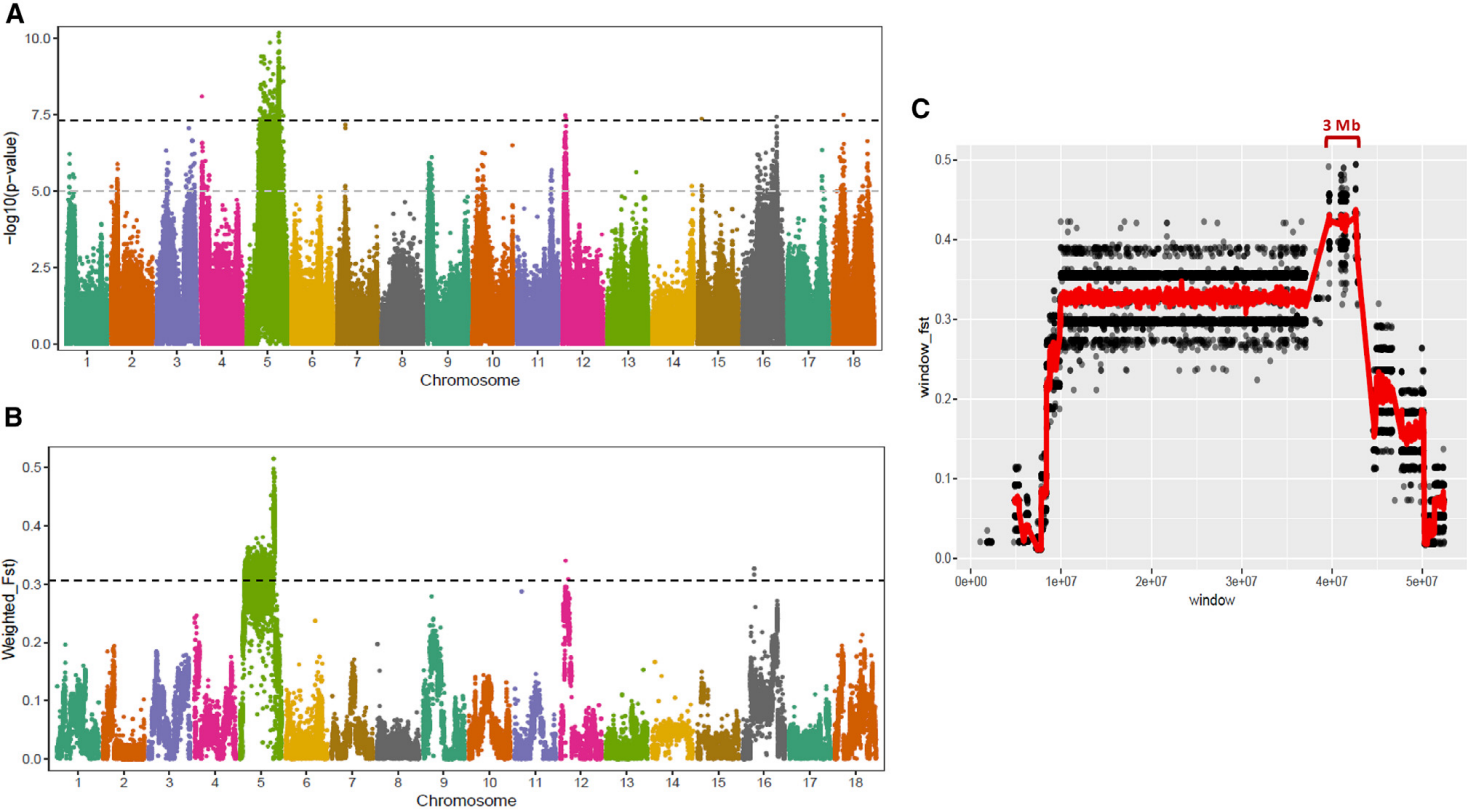
GWAS (A) Manhattan plot showing genomic regions associated with snail resistance in the RIL population. Fisher’s exact test (−log10(*p* value)) was used to investigate the association between the resistant phenotype and SNPs (*n* = 7,330,259). The bold dark dashed line and gray dashed line indicate the genome-wide significance levels at *p* = 5 × 10^−8^ and *p* = 1 × 10^−5^, respectively. (B) Genetic divergence test (F_ST_) between resistant and susceptible snails in 10-kb windows (*n* = 51,284) for variants across the 18 chromosomes. The bold dark dashed line indicates significant genome-wide F_ST_ at *p* ≤ 0.05 for each chromosome. (C) Sliding window-based F_ST_ analysis on chromosome 5. Individual variants are represented by gray circles (displaying only those with F_ST_ > 0.01, *n* = 74,499), while mean values are shown using sliding windows of 10-kb, marked by red lines. The blue bracket indicates a ∼3 Mb genomic region (positions: 39,634,500 nt–42,686,436 nt) with the highest divergence. Data are represented as mean ± SEM.

GWAS was performed on the RIL population using 7,330,259 SNP markers. A total of 120,698 SNPs were identified at a significant level of *p* ≤ 1 × 10^−5^. Among these, 837 SNPs were found to have significant associations (*p* ≤ 5 × 10^−8^) and were distributed across six chromosomes (chr 4, 5, 12, 15, 16, and 18). However, most of the SNPs (99.28%, 830/836) were located on chromosome 5 (Figure 3A; Table S3).

Fixation index F_ST_ analysis of the 7,330,259 SNP markers was performed using a 10-kb window analysis on susceptible and resistant RIL groups. This approach allowed us to identify regions that showed differences between the two groups. A jackknife procedure was used to test whether F_ST_ values were statistically different from zero.^53^ We used a significance level of *p* ≤ 0.05, with a weighted F_ST_ value of 0.3061 from the genome-wide distribution, to define high F_ST_ outliers. Out of the 51,284 10-kb windows (F_ST_ > 0), a total of 2,568 10-kb windows showed significant divergence between the two phenotypes. Among these, 2,563 10-kb windows were located on chromosome 5, two on chromosome 12, and three on chromosome 16 (Figure 3B; Table S4).

From the genome-wide 7,330,259 SNP markers, 470,229 SNPs on chromosome 5 were extracted and filtered to exclude SNPs with a significant deviation from the 1:1 segregation ratio (*p* < 0.01) and low homozygous genotypes (≤40). This resulted in 74,499 SNPs at F_ST_ > 0.01, including 42,473 SNPs at F_ST_ > 0.3061 and 373 SNPs with the highest F_ST_ value (>0.433) in a small genomic region (39.6–42.6 Mb) (Table S5). Similar filtering criteria were applied to chromosomes 12 and 16, resulting in 16 significant SNPs on chromosome 12 and 5 significant SNPs on chromosome 16 (Table S6). Overall, this analysis revealed a ∼3 Mb region (position: 39,634,500 nucleotides [nt]–42,686,436 nt) on chromosome 5 that exhibited the highest divergence in 10-kb windows between the susceptible and resistant RIL groups (Figure 3C). The average F_ST_ value of the specific 3 Mb region (0.41 ± 0.004) was significantly greater than that of the neighboring 3 Mb region to the left (0.29 ± 0.004), as determined by a pooled t test (*t*_458_ = 19.5, *p* < 0.0001). We designated this genomic region or QTL as the *B. glabrata* schistosome resistance region 1 (BgSRR1).

### Bin marker identification and recombination pattern analysis

**Figure 4:**
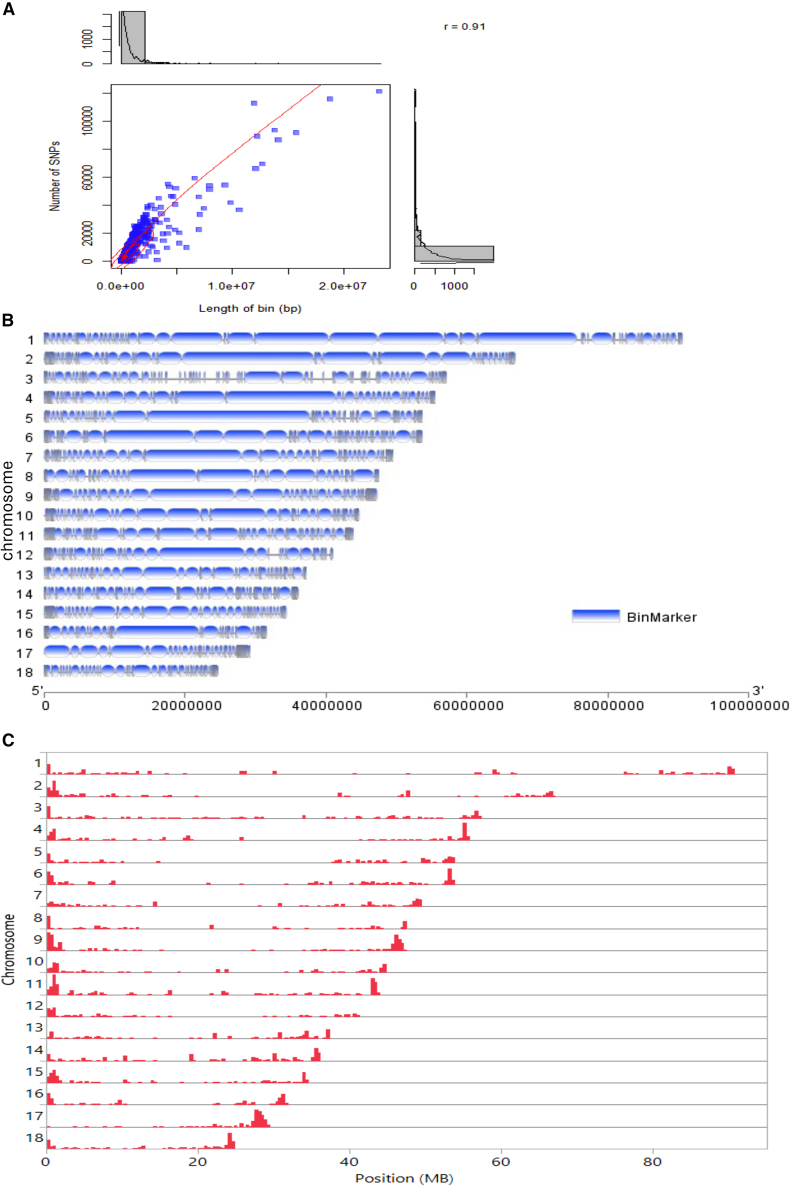
Bin marker analysis (A) Scatterplot of bin markers, with the x axis indicating the length of the bin markers and the y axis indicating the number of SNPs per bin. Red dashed lines represent the linear trend line. Subplots on the top and right display histograms of bin length and the number of SNPs in each bin marker. Data are represented as mean ± SEM. (B) Distribution of bin markers across the 18 chromosomes. (C) Distribution of genome-wide recombination breakpoints on each of the 18 chromosomes. The red bar shows the relative number of observed recombination crossover sites in the RIL population.

Further analysis was conducted using bin marker-based genetic mapping.^54^^,^^55^ To identify genomic intervals without recombination events in RIL populations, a 10-kb sliding window with Binmarker v.2.3 was used to generate a total of 2,190 bin markers from the 7,330,259 SNPs across the 18 chromosomes (Table S7). The length of the bins was found to be correlated with the number of SNPs per bin (r = 0.91) (Figure 4A). On average, the length of a bin was 353,427 bp, and each bin contained an average of 3,347 SNPs (Figure 4B). Analysis of the distribution of the genome-wide recombination pattern revealed that the centromeric regions of most chromosomes had significantly fewer recombination events (Figure 4C).

### QTL analysis of snail resistance to schistosomes

**Figure 5:**
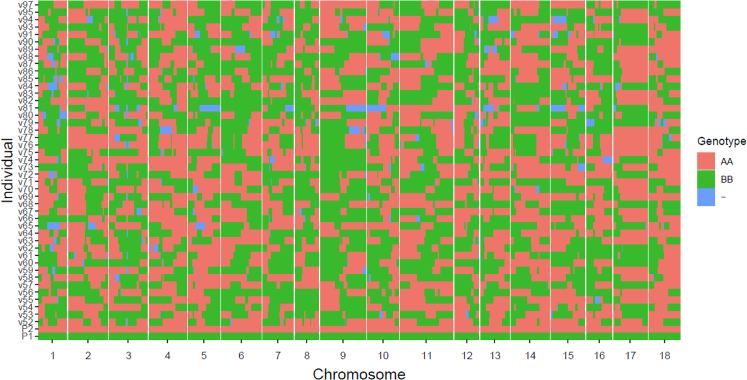
Recombination bin map of the 46 RILs The colors red, green, and blue represent the genotypes of the iM line (AA), the iBS90 (BB), and the heterozygous genotype (AB) and missing data, respectively. P1 and P2 refer to the iM line and iBS90, respectively.

To further refine our analysis, we filtered 2,190 bin markers by excluding those with a significant deviation (*p* < 0.001) from the 1:1 segregation ratio. The remaining markers were then used to construct a linkage map and conduct QTL analysis. A total of 2,121 bin markers were used to construct the genetic map, resulting in a map distance of 1,311.4 cM, with an average distance of 0.62 cM between adjacent markers (Figure 5). The number of bin markers varied across chromosomes, ranging from 81 on chromosome 8 to 182 on chromosome 11. The largest marker gap was observed on chromosome 1 with a length of 15.47 cM, followed by chromosomes 4, 8, and 12, each with gaps of approximately 13 cM (Table S8).

**Figure 6:**
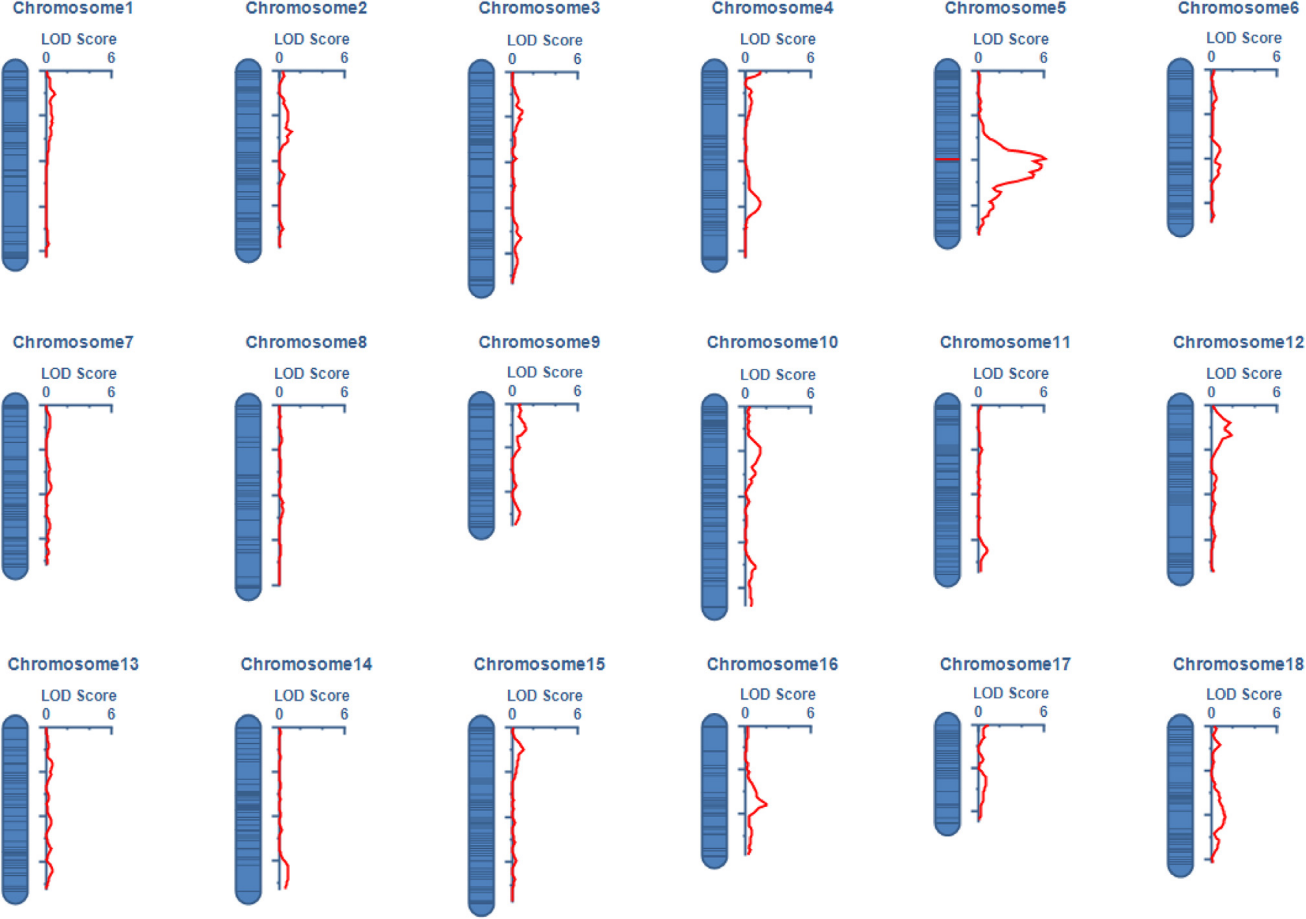
QTL profiling of schistosome resistance across the 18 chromosomes of the snail RILs A major QTL was detected on chromosome 5 between bin markers BgChr5_39634500:1:1 and BgChr5_39675885:1157240:1117, with a significant LOD score of 6.04.

QTL analysis indicated that snail resistance is controlled by a major QTL on chromosome 5, located between bin marker BgChr5_39634500:1:1 and BgChr5_39675885:1157240:1117, with a significant logarithm of odds (LOD) score of 6.04 (Figure 6). The bin marker on the left contains only one SNP at position 39,634,500 nt on chromosome 5, while the bin marker on the right encompasses 1,117 SNPs and spans a physical length of 1,157,240 bp. This QTL has an additive effect of 0.35 and accounts for 46.2% of the phenotypic variance. The 95% confidence interval for this QTL ranges from 37.5 cM to 39.5 cM, corresponding to a physical position from 39.6 to 41.9 Mb on chromosome 5, further confirming the 3 Mb region identified as the region of highest F_ST_ divergence (Figure 3C).

### Gene identification and GO analysis

A total of 118 protein-coding genes were identified in the BgSRR1, and detailed information about all 118 genes can be found in Table S9. Among the 118 genes, 73 have homologs with known functions, which are listed in Table 1 (see the discussion section for more information).

**Table 1:** A list of genes in BgSRR1 that encode proteins with homologs

No	Gene product	No	Gene product	No	Gene product
1	*cis*-aconitate decarboxylase	26	FGGY carbohydrate kinase domain-containing protein	51	trichohyalin-like isoform X1
2	thiosulfate sulfurtransferase	27	ran-binding protein 3	52	splicing factor 3B subunit 5
3	trichohyalin	28	glutathione peroxidase 1	53	TATA box-binding protein-like protein 1
4	WASH complex subunit strumpellin	29	DNA-directed RNA polymerases I, II, and III subunit RPABC1	54	acyl-protein thioesterase 1-like isoform X2
5	exportin-2	30	phosphatidylinositol-glycan-specific phospholipase D	55	NIPA-like protein 2
6	CDK5 and ABL1 enzyme substrate 1-like isoform X1	31	bifunctional polynucleotide phosphatase/kinase	56	transmembrane protein 59
7	apolipophorins-like isoform X1	32	serine-rich adhesin for platelets-like isoform X1	57	D-amino-acid oxidase (2)
8	transmembrane protein 8A-like isoform X1	33	annexin A4	58	kinesin-like protein KIF2A
9	calcium homeostasis endoplasmic reticulum protein	34	protein phosphatase 1 regulatory subunit 7	59	tachykinin-like peptides receptor 99D
10	MAP kinase-interacting serine/threonine-protein kinase 1	35	orexin receptor type 2 (2)	60	BgFReDn19 (4)
11	DNA repair endonuclease XPF-like isoform X1	36	adipocyte plasma membrane-associated protein (4)	61	protein FAM166C A
12	superkiller viralicidic activity 2-like 2 isoform X1	37	E3 ubiquitin-protein ligase HECTD3	62	transcription elongation factor A N-terminal and central domain-containing protein 2-like isoform X1
13	derriere protein	38	ankyrin repeat domain-containing protein 13C (2)	63	mediator of RNA polymerase II transcription subunit 26
14	mRNA export factor	39	ethylmalonyl-CoA decarboxylase-like isoform X2	64	SCL-interrupting locus protein
15	ATP synthase subunit delta mitochondrial	40	transmembrane protein 65 (2)	65	cytidine monophosphate (UMP-CMP) kinase 1 cytosolic
16	diisopropyl-fluorophosphatase	41	zinc-finger protein 451	66	choline transporter-like protein 1
17	acyl-CoA synthetase YngI	42	mediator of DNA damage checkpoint protein 1	67	high-affinity cAMP-specific 3p 5′-cyclic phosphodiesterase 7A
18	DNA-directed RNA polymerase I subunit RPA1	43	defense protein 3 (3)	68	mitochondrial fission regulator 2
19	N-acetyltransferase ESCO1	44	ferric-chelate reductase 1	69	pikachurin
20	ATP-dependent RNA helicase DDX43	45	DNA excision repair protein ERCC-6-like 2	70	histone H2A
21	chromatin modification-related protein EAF7	46	TBC1 domain family member 2B (3)	71	THAP domain-containing protein 6
22	stromal membrane-associated protein 1-like isoform X1	47	leucine-rich repeat-containing protein	72	galectin-4
23	zinc-finger SWIM domain-containing protein 5-like isoform X1	48	protein phosphatase 1 regulatory subunit 7	73	eyes absent 4
24	GTP-binding protein Di-Ras2	49	peroxidasin	–	–
25	HMG box-containing protein 4	50	regulator of telomere elongation helicase 1 isoform X1	–	–

Note: The number in parentheses at the end of a gene name is the total number of the genes from the same gene family (with the same gene name) in the BgSRR1.

**Figure 7:**
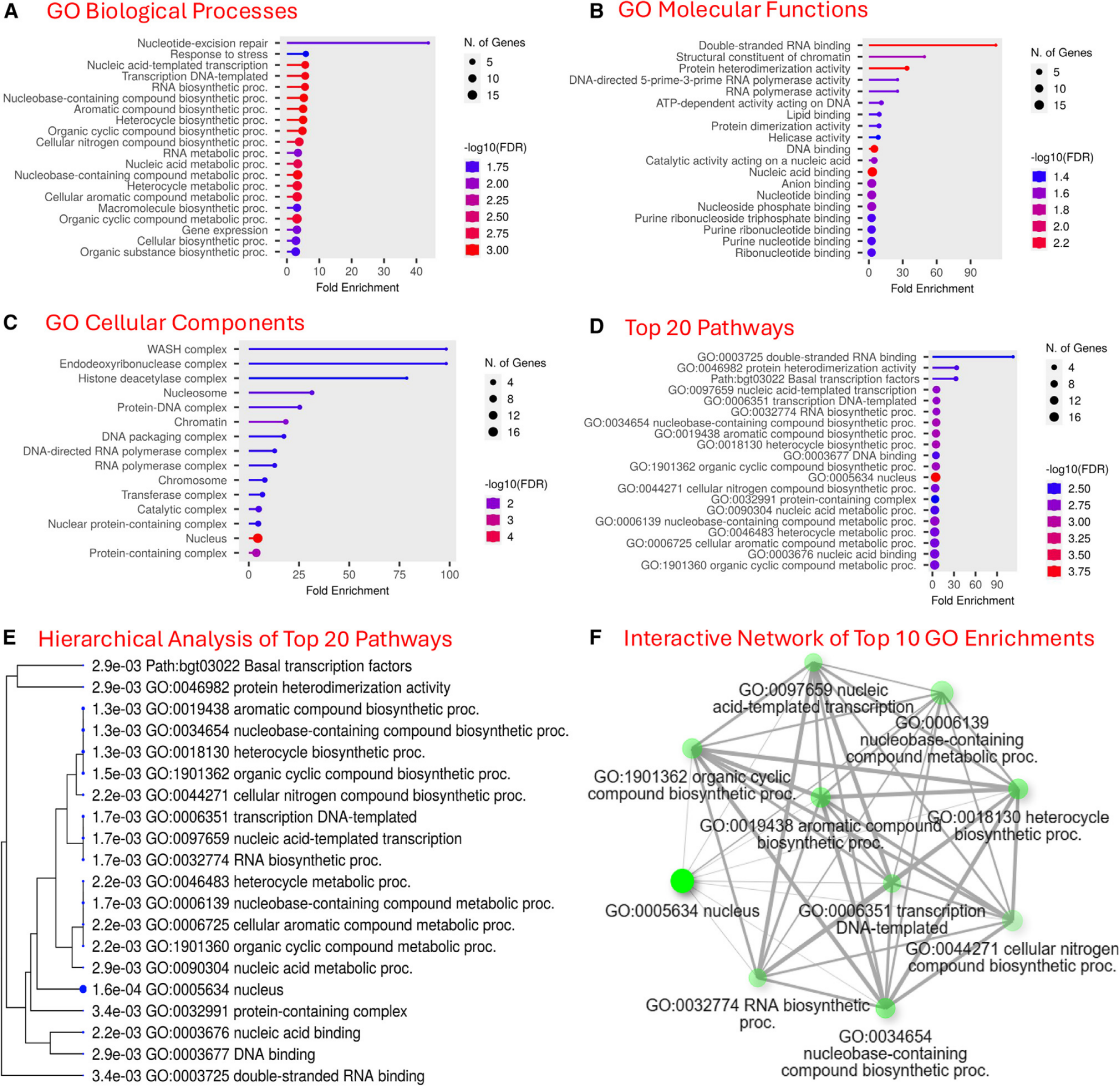
GO and KEGG pathway analyses (A–D) show biological processes, molecular functions, cellular components, and the top 20 pathways, respectively. (E) The hierarchical clustering tree summarizes the correlation among the top 20 significant pathways. Pathways with many shared genes are clustered together. Larger dots indicate more significant *p* values. (F) The interactive network plot shows the relationship between the top 10 enriched pathways. Two pathways (nodes) are connected if they share 20% (default) or more genes. Darker nodes represent more significantly enriched gene sets. Larger nodes represent larger gene sets. Thicker edges represent more overlapping genes.

Gene Ontology (GO) analysis shows enriched biological processes, molecular functions, cellular components, and pathways (Figures 7A–7E). Three significant Kyoto Encyclopedia of Genes and Genomes (KEGG) pathways associated with double-stranded RNA binding, protein heterodimerization activity, and basal transcription factors, along with 68 enriched GO categories, were revealed. These GO categories comprise 21 biological processes, 15 cellular components, and 32 molecular functions (Table S10). The most highly enriched categories include double-stranded RNA binding for molecular function, nucleotide excision repair for biological processes, and three cellular components (WASH complex, endodeoxyribonuclease complex, and histone deacetylase complex). The functional linkage network analysis revealed the top 10 GO enrichments, including GO:0005634 nucleus, GO:0034654 nucleobase-containing compound biosynthetic process, GO:0019438 aromatic compound biosynthetic process, GO:0018130 heterocycle biosynthetic process, GO:1901362 organic cyclic compound biosynthetic process, GO:0097659 nucleic acid-templated transcription, GO:0006351 transcription DNA-templated, GO:0032774 RNA biosynthetic process, GO:0006139 nucleobase-containing compound metabolic process, and GO:0044271 cellular nitrogen compound biosynthetic process (Figure 7F).

## Discussion

A thorough understanding of the fundamental mechanisms that control the traits of interest is crucial for the development of genetically modified organisms for biomedical research. Extensive genetic analyses have been conducted on anti-parasite traits in disease vectors such as mosquitoes,^56^^,^^57^^,^^58^^,^^59^^,^^60^ leading to active studies on genomic modifications for both basic and applied research.^38^^,^^39^^,^^40^ However, limited progress has been made with schistosomiasis vector snails, hindering our ability to pursue similar innovative work for schistosomiasis control. Different from the relevant genetic studies conducted on vector snails or other mollusks, the current study is based on our long-term efforts to develop genetic resources (the homozygous iM line and iBS90, F2 segregating population, and RILs) and genomic resources (scaffold- and chromosome-level assembled genomes) for the schistosomiasis model snail *B. glabrata*, with the main objective of deciphering the mechanisms underlying snail resistance to schistosomes.^45^^,^^52^

The development of RIL snails represents one of our efforts in this direction, despite the painstaking nature of the work involved. RIL snails were produced using an advanced design. The RILs were obtained through two crosses: a parental outcross and an F2 intercross, followed by 10 generations of selfing (Figure 1). This design differs from the standard RIL design, which involves 6–7 generations of inbreeding starting with F2 offspring resulting from a single outcross (parental cross).^49^^,^^51^^,^^61^ The increased intercrossing, along with a greater number of generations of inbreeding (selfing), should further enhance mapping resolution and reduce the size of QTL by accumulating additional meiotic crossover events.

Indeed, the resistance QTL size was reduced from approximately 8 Mb using the F2 population to about 3 Mb, as revealed by the current RIL population. Importantly, our current findings from two genetic analyses, GWAS and bin marker-based QTL, unequivocally confirm the 3 Mb resistance locus BgSRR1 and its genomic location on chromosome 5, which are also consistent with our previous F2-ddRADseq mapping^45^ (see further discussion in the following).

We carefully determined the resistance phenotype of each RI line, as it is important for genetic mapping. In our genetic studies, resistance or susceptibility was defined based on cercarial shedding rather than snail infection. Cercarial shedding directly contributes to disease transmission and human infection. After exposure to schistosome miracidia, many snails become infected or are penetrated by the miracidia, but not all infected snails shed cercariae. Some parasites experience impeded development but still survive in the snail host for a long time, resulting in no cercariae being released from these hosts. This phenomenon was observed in our recent work, which showed that some resistant snails (without shedding cercariae) possessed a varying number of schistosome reads from DNA extracted from the entire snail body.^45^ The laboratory-based finding was confirmed by field observations. A large-scale polymerase chain reaction (PCR)-based surveillance program in coastal Kenya demonstrated that the rates of schistosomes present in snail hosts, as detected by PCR, were significantly higher than those observed through cercarial shedding (28%–54% vs. 0.14%–3.4%).^62^ If a snail does not shed cercariae, it plays no role in disease transmission, regardless of whether it is infected. As our goal is to apply our findings to field applications, the focus of our investigations is on the phenotype of cercarial shedding rather than on infections.

We employed WGS as a genotyping assay to sequence the genomes of individual RIL snails instead of using pooled DNA samples from multiple snails with the same phenotype (Pool-seq). For each RIL, deep genome sequencing (∼16X coverage) was conducted on a single RIL snail to represent the corresponding RIL for genetic mapping, as all individuals within the same RIL are nearly genetically identical.^49^^,^^50^^,^^51^ Although this approach is more costly and labor intensive compared to Pool-seq, it provides data that can be used for accurate analyses or re-analyses. As a result, we were able to identify a large number of SNPs (*n* = 7,330,259) and bin markers (*n* = 2,190) across the 46 RIL genomes. The genetic mapping conducted with the current linkage map, which has denser markers compared to the F2-based linkage map (0.62 cM vs. 1.73 cM), has revealed a smaller QTL size. Subsequent GWAS and QTL analysis both indicated that the snail *B. glabrata* has a ∼3 Mb BgSRR1 on chromosome 5. This genomic region shows a peak F_ST_ value across chromosome 5 (Figure 3C). Linkage mapping analysis of this region reveals only three recombination points, identifying a large haplotype block spanning approximately 1.16 MB and containing 1,117 SNPs.

**Figure 8:**
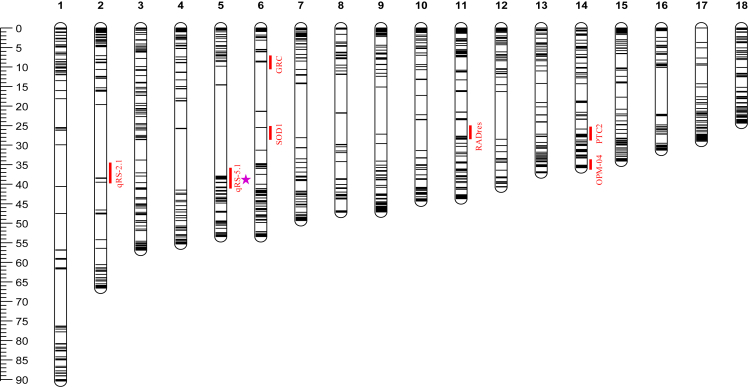
Distribution of QTLs reported on the 18 chromosomes based on the chromosome-level assembled genome of iM line *B. glabrata* The previously reported QTLs, including OPM-0423,^41^ GRC,^42^ RADres and SOD,^43^ PTC2,^44^^,^^46^ and qRS2.1 and qRS5.1,^45^ are indicated by solid red bars. The pink star marks the chromosomal location of BgSRR1. The RAPD marker OPZ-11^41^ is not indicated due to its repetitive sequence.

BgSRR1 identified by the current RIL-WGS approach agrees with our previous findings using the F2-ddRADseq analysis (i.e., the resistance locus on chromosome 5).^45^ In addition to chromosome 5, SNPs linked to resistance were also detected on chromosomes 4, 12, 15, 16, and 18, despite the limited number of SNPs. These chromosomes, especially chromosomes 12 and 16, deserve attention in future studies. We cannot exclude the possibility that loci on these chromosomes may have an effect or a minor effect on resistance. To compare our current findings with previous reports, we mapped QTLs reported by other laboratories^41^^,^^42^^,^^43^^,^^44^^,^^46^ to the 18 chromosomes based on our chromosome-level assembly of *B. glabrata*.^52^ Surprisingly, the chromosomes containing QTLs identified by other research groups differ from chromosome 5 and from those with a limited number of significant SNPs (i.e., chromosomes 4, 12, 15, 16, and 18) (see Figure 8). Please note that the chromosome numbers (i.e., the order from 1 to 18) in Figure 8 from Zhong et al.^52^ are not exactly the same as the linkage group numbers in Figure 7 of the paper published by Bu. et al..^45^ Therefore, the genes identified by other groups, including a cluster of genes encoding transmembrane proteins,^41^^,^^42^^,^^43^^,^^44^^,^^46^ are not present in our BgSRR1. The reason for the discrepancy between our findings—both previous and current—and those reported by others is still unknown.

Although the GO analysis of the protein-coding genes in BgSRR1 was conducted, caution is warranted in interpreting the findings because about one-third of the genes in the region lack homologs with known functions and could not be included in the GO analysis. Nonetheless, the analysis offers useful insights into the mechanisms of schistosome resistance in snails. Some previously unrecognized pathways may be involved in the defense responses. For example, the enriched GO categories include many genes and pathways related to metabolism. Immunometabolism has recently emerged as a dynamic field in immunology but has not yet been explored in snail immunology or host-parasite interactions.^63^^,^^64^^,^^65^

Focusing on genes with known functions, we are excited to find that BgSRR1 possesses genes known to be involved in cellular immunity in snails. The presence of the *mitogen-activated protein kinase* (MAPK) gene in BgSRR1 is supported by earlier studies suggesting that MAPK-based signal transduction plays a critical role in hemocyte-mediated encapsulation and H_2_O_2_ production, leading to the killing of intramolluscan schistosomes.^66^^,^^67^^,^^68^ Interestingly, two genes from the *peroxidase* gene family, *glutathione peroxidase* and *peroxidase*, have been identified in BgSRR1. Peroxidases are antioxidative enzymes that scavenge H_2_O_2_ and inhibit apoptosis.^69^ An earlier study also revealed that a *thioredoxin peroxidase* or *peroxiredoxin* gene was highly expressed in resistant *B. glabrata* snails compared to susceptible ones in response to schistosome infection.^70^ Additionally, BgSRR1 contains a gene encoding the enzyme thiosulfate sulfurtransferase, which may also have antioxidative properties.^71^ These findings suggest that BgSRR1 is involved in regulating cell-mediated immunity, particularly in relation to redox balance.

BgSRR1, however, does not contain genes previously reported to play a significant role in humoral immunity in *B. glabrata*, such as *biomphalysin*,^72^ *fibrinogen-related proteins* (*FREPs*),^73^^,^^74^^,^^75^^,^^76^^,^^77^ *macrophage migration inhibitory factor* genes,^78^ and genes encoding proteins associated with the Toll-like receptor pathway.^79^^,^^80^ Notably, we have identified other humoral immune genes in BgSRR1, including *apolipophorin*,^81^ *defense proteins*,^82^ *RNA helicase*,^83^ and *E3 ubiquitin protein ligase* genes,^84^ although the roles of these genes in schistosome resistance have not been reported in snails. It is worth mentioning that BgSRR1 contains four *ficolin* genes (only fibrinogen [FBG]) but none of the *FREPs*. Ficolins, which are important players in innate immunity, have been extensively studied in the mosquito-*Plasmodium* model,^85^^,^^86^^,^^87^ but not in the snail-schistosome system.

We have observed genes in BgSRR1 that encode enzymes involved in immune cell metabolism. For example, the enzyme *cis*-aconitate decarboxylase, encoded by *immune response gene 1*, produces itaconate, an intermediate metabolite from the tricarboxylic acid cycle in immune cells.^88^ Recent studies have demonstrated that itaconate is an important immunometabolite that regulates host defense and inflammation.^89^^,^^90^ The potential role of immunometabolism in defense is also suggested in the GO analyses discussed earlier. Future investigations into the functions of these genes in BgSRR1, especially those not yet studied in *B. glabrata*, may reveal unexpected aspects of snail-parasite interactions, provide valuable insights into snail defenses, and help identify key resistant genes.

In conclusion, our approach (RIL-WGS), combined with our RIL genetic resource, powerful genome-wide genotyping, GWAS, and bin marker-assisted QTL analysis, has enabled the identification of the BgSRR1 on chromosome 5 of *B. glabrata*, an important molluscan vector of human schistosomiasis. The identification of BgSRR1 and the genes conferring schistosome resistance has the potential to advance our understanding of host-parasite interactions and facilitate the development of snail-targeted biocontrol strategies for schistosomiasis, a parasitic disease that infects 251 million people worldwide.^91^

### Limitations of the study

While our current findings are robust and supported by a well-developed genetic resource, reliable phenotype and genotype data, and multiple genetic analyses, it is important to acknowledge their limitations, which are common in genetic studies. Firstly, the use of RILs limits the ability to capture information regarding dominance due to their high homozygosity.^92^ In fact, our RIL-WGS approach did not detect the presence of a susceptibility-associated QTL under the dominance effect on chromosome 2.^45^ Secondly, it should be recognized that our findings are based on a well-developed laboratory system. Therefore, further evaluation and testing of our findings in other systems, particularly in field settings, are necessary.

## Resource availability

### Lead contact

Further information and requests for resources and reagents should be directed to and will be fulfilled by the lead contact, Si-Ming Zhang (zhangsm@unm.edu).

### Materials availability

This study did not generate any new unique reagents.

### Data and code availability

• The Illumina sequence data generated from 46 RIL snails have been deposited at the National Center for Biotechnology Information (NCBI) and are publicly available as of the date of publication. Accession numbers are listed in the key resources table.
• This paper does not report original code.
• Any additional information required to reanalyze the data reported in this paper is available from the lead contact upon request.

## Acknowledgments

This study was funded by the 10.13039/100000002National Institutes of Health (NIH) (https://www.nih.gov) grant R01 AI170587 to S.-M.Z. The funder had no role in the study design, data collection and analysis, decision to publish, or preparation of the manuscript. The PR1 strain of *Schistosoma mansoni* was provided by the NIAID Schistosomiasis Resource Center of the Biomedical Research Institute (Rockville, MD, USA) through NIH-NIAID Contract HHSN272201700014I.

## Author contributions

S.-M.Z.: conceptualization, breeding of RIL snails and testing of their phenotypes, DNA extraction and quality evaluation, investigation, writing – original draft, and writing – review and editing; G.Y.: investigation and writing – review and editing; A.L.: writing – review and editing; D.Z.: data analysis, investigation, writing – original draft, and writing – review and editing. All authors read and approved the final manuscript.

## Declaration of interests

The authors declare no competing interests.

## STAR★Methods

### Key resources table

**Table:** 

REAGENT or RESOURCE	SOURCE	IDENTIFIER
**Chemicals, peptides, and recombinant proteins**		

CTAB solution	Teknova	Lot no: C219009G1801
Chloroform: Isoamyl alcohol	Sigma-Aldrich	Lot no: 1003577830
Proteinase K	TermoScientific	Lot no: 10198999
RNase A	TermoScientific	Lot no: 2653498
Isopropyl alcohol	Honeywell	Lot no: CZ999
200 proof pure ethanol	KOPTEC	Lot no: A08232309D

**Deposited data**		

Illumina sequence data	National Center for Biotechnology Information (NCBI) (https://www.ncbi.nlm.nih.gov)	BioProject ID: PRJNA1133633. BioSample accession numbers: SAMN42382410-SAMN42382455.

**Experimental models: Organisms/strains**		

iM line of *Biomphalaria glabrata*	University of New Mexico, USA	Si-Ming Zhang
iBS90 of *Biomphalaria glabrata*	University of New Mexico, USA	Si-Ming Zhang
RILs of *Biomphalaria glabrata*	University of New Mexico, USA	Si-Ming Zhang
PR1 strain of *Schistosoma mansoni*	Biomedical Research Institute in Maryland, USA	Margaret Mentink-Kane

**Software and algorithms**		

Trimmomatic 3.9	Bolger et al.^93^	https://github.com/timflutre/trimmomatic
CLC Genomics Workbench 23	QIAGEN Aarhus, Denmark	https://digitalinsights.qiagen.com/
bcftools	Danecek et al.^94^	https://samtools.github.io/bcftools/bcftools.html
vcftools	Danecek et al.^95^	https://vcftools.github.io/index.html
PLINK 1.9	Purcell et al.^96^	https://www.cog-genomics.org/plink/
JMP 14	SAS Institute Inc., Cary, NC	https://www.jmp.com/en_us/home.html
Binmarkers-v2	Qin et al.^97^	https://github.com/lileiting/Binmarkers-v2
IciMapping	Meng et al.^98^	http://www.isbreeding.net
ShinyGO	Ge et al.^99^	https://bioinformatics.sdstate.edu/go/

**Other**		

A scaffold-level assembled genome of iM line and iBS90 of *Biomphalaria glabrata*	Bu et al.^45^	https://doi.org/10.1038/s42003-022-03844-5
A chromosome-level assembled genome sequence of *Biomphalaria glabrata*	Zhong et al.^52^	https://doi.org/10.1038/s42003-022-03844-5

### Experimental model and study participant details

#### Snails and schistosomes

The snail *Biomphalaria glabrata*, a major intermediate host of human schistosomiasis in Neotropical countries, was used for this study. The efforts to generate *B. glabrata* recombinant inbred lines (RILs) are described in the results section. Breeding, cultivation, and storage of the RIL snails were conducted at the Center for Evolutionary and Theoretical Immunology (CETI), University of New Mexico (UNM), United States. The generation of recombinant inbred (RI) lines (RILs) is also described in the results section. The PR1 strain of *Schistosoma mansoni* used to infect the snails was collected from the Biomedical Research Institute in Maryland, USA (https://www.afbr-bri.org).

### Method details

#### Exposure of schistosome miracidia to snails

To determine the phenotype of the RI lines, 6–8 juvenile snails (0.3–0.6 mm shell diameter) were randomly chosen from each RI line for infection. The snails were individually placed into the wells of a 24-well cell culture plate (one snail per well) and 20 schistosome miracidia were added to each well. The snails were fully submerged in water overnight to ensure complete exposure to the miracidia. Afterward, the exposed snails were transferred to large tanks for continued culturing until cercarial shedding was performed.

#### Determination of schistosome resistance phenotype

Examination of the phenotype began at 45 days post-exposure (dpe). The exposed snails were placed individually in the wells of a 24-well plate and exposed to light for 0.5 h (hr). Snails that shed cercariae were considered susceptible to schistosomes, while those that did not shed cercariae were transferred to the aquatic tank and cultured for later examination of shedding. If a snail did not shed cercariae at 60 dpe, it was classified as a resistant snail. This procedure allowed us to determine the phenotype of each RI line. Once the phenotype was determined, the remaining snails from each RI line (those not exposed to schistosomes) were preserved in liquid nitrogen for DNA extraction. Only the RI lines that exhibited the same phenotype in all tested individuals were selected for genetic mapping.

#### DNA extraction

A single snail thawed from liquid nitrogen was placed into a 1.5 mL tube and ground in 750 μL of CTAB buffer.^100^ After homogenization, 20 μL of proteinase K (20 μg/μL) was added to the homogenate and incubated at 60°C for 1 h (hr). Next, 750 μL of chloroform: isoamyl alcohol (24 : 1) was added and rocked for 0.5 h. Following centrifugation, the supernatant was transferred to a new tube. To degrade and remove RNA, 10 μL of RNase (10 μg/μL) was added to the new tube and incubated at 37°C for 0.5 h. An equal volume (750 μL) of chloroform: isoamyl alcohol was added to the solution and rocked at room temperature for 10 min (min). Genomic DNA was precipitated using isopropyl alcohol, washed with 70% ethanol, and dissolved in nuclease-free water.

#### Library preparation and WGS

The genomic DNA was qualified and quantified using agarose gel electrophoresis (1%) and the Qubit 2.0 DNA HS Assay (ThermoFisher), respectively. For library preparation, the KAPA Hyper Prep kit (Roche) was used. In brief, the genomic DNA was sheared into 500 bp fragments using the Covaris LE220-plus. After ligating the adapters, the fragments were amplified by PCR. The quantity and quality of the libraries were assessed using the Qubit 2.0 DNA HS Assay, the Tapestation High Sensitivity D1000 Assay (Agilent Technologies), and the QuantStudio 5 System (Applied Biosystems). Finally, the libraries were sequenced using an Illumina NovaSeq S4.

#### Trimming, mapping reads, and SNP calling

The raw Illumina reads for the two parental lines (iM line and iBS90) were retrieved from GenBank (accession number: SRR16289947 for the iM line and SRR16289905 for the iBS90).^45^ It is important to note that all Illumina data, including those from the two parental snails and the RIL snails were generated from the same Illumina platform (Admera Health; www.admerahealth.com) and the same quality control was applied to all samples. All raw reads were trimmed and cleaned using Trimmomatic v0.39^93^ with the following parameters: ‘ILLUMINACLIP: TruSeq3-PE-2.fa: 2:30:10 HEADCROP:7 LEADING: 25 TRAILING: 25 SLIDINGWINDOW: 4:25 MINLEN: 36’. The clean reads from each RIL sample were individually aligned to the reference genome of *B. glabrata* (GenBank assembly GCA_025434175.2)^52^ using the Map Reads to Reference tool in the QIAGEN CLC Genomics Workbench 23 (Qiagen Genomics, Denmark) with the default parameters. SNP calling for the iM line and iBS90 was conducted according to the methods described previously.^45^ The Identify Known Mutations from Mappings tool of the CLC workbench was used to genotype the RILs individually at SNP loci that were polymorphic in the two parental snails. Subsequently, the SNPs identified from the CLC were exported individually to VCF files and then merged into a single VCF file using bcftools.^94^ Finally, the resulting VCF file was filtered using vcftools^95^ with the following parameters: ‘--minDP 5; --maxDP 100; --maf 0.1; --max-missing 0.8'.

#### Genome-wide SNP genotyping and GWAS

Single SNP genotype and phenotype association analyses were conducted using PLINK software.^96^ To identify significant associations, we applied the widely accepted threshold of *p* < 5 × 10^−8,^^101^ which is derived from a Bonferroni correction for all independent SNPs in the genome. To measure the divergence between susceptible and resistant populations, we calculated the fixation index (F_ST_) using VCF tools. We used a sliding window of 10 kb, with an increment of 5 kb, to perform this calculation. Significant high F_ST_ outliers were identified based on the 95th quantile from the genome-wide distribution and jackknife procedure. Outlier analysis was conducted using the Jackknife Distances in SAS JMP 14. This involved calculating pairwise distances between data points, resampling the data by removing one point at a time, and analyzing the variance of the resulting distances. Points with unusually high variance were flagged as potential outliers and further validated.

#### Genetic bin marker calling

To identify genomic intervals in a mapping population with no recombination events, we used the Binmarker-v2.3 tool (https://github.com/lileiting/Binmarkers-v2).^97^ This tool employs a sliding window approach of 10 kb to generate genetic bin markers. Missed genotypes were imputed and miscoded genotypes were corrected using strict criteria: a genotype that differed from surrounding genotypes, no missing data in surrounding genotypes, and identical surrounding genotypes. Next, markers with 100% identical markers were merged together. These bin markers were then organized based on the physical position of the chromosome. A change in genotyping within any sample was considered a recombination breakpoint. SNPs between recombination breakpoints were classified as bin markers, indicating that no recombination occurred within that bin.

#### Construction of the linkage map and QTL analysis

Bin markers showing significant deviation (*p* < 0.001) from the 1:1 segregation ratio were excluded from constructing the linkage map. Heterozygous genotypes were treated as missing data and imputed using the "maxmarginal" method implemented in the R/qtl package. Linkage map construction and QTL analysis were conducted using QTL IciMapping version 4.2.53.^98^ Simple interval mapping and inclusive composite interval mapping were employed to detect potential QTLs associated with snail resistance or susceptibility to schistosome parasites. A significant threshold of the logarithm of odds (LOD) (LOD = 4.0) based on 1,000 permutation tests was applied.

#### Analysis of protein-coding genes in the QTL region

The coding genes were further verified manually by BLAST searching against NCBI databases. GO (Gene Ontology) and KEGG (Kyoto Encyclopedia of Genes and Genomes) analyses were performed using the web-based tool ShinyGO 0.80 (http://bioinformatics.sdstate.edu/go/).^99^ A flowchart showing bioinformatic and genetic analyses is provided in Figure S1.

### Quantification and statistical analysis

PLINK was employed for genome-wide association studies (GWASs) with a stringent *p*-value threshold of 5 × 10^−8^ to identify significant associations. Vcftools was used to calculate fixation index (F_ST_) statistics, and outlier analysis was performed using Jackknife Distances in SAS JMP 14, with a *p*-value threshold of <0.05 for significance. A permutation test with 1,000 iterations and a type I error rate of 0.05 was used to establish the significance threshold for QTL LOD scores. Functional enrichment analysis, including GO and KEGG pathways, was conducted with an E-value threshold of < 1e-5. Significant GO term enrichment was assessed using Fisher’s exact test, applying a *p*-value threshold of <0.05. These analyses identified genetic variants associated with snail resistance and explored their functional implications.

## Supplemental information

Document S1. Figure S1

Table S1. Summary of read trimming and read mapping for the 46 RIL snails and their parents, related to Figure 2A

Table S2. Number of SNPs and coverage depth of the RIL population, related to Figures 2B and 2C

Table S3. List of SNPs significantly associated with the resistant phenotype, related to Figure 3A

Table S4. Sliding-window analysis of divergence in two RIL phenotypes (FST), related to Figure 3B

Table S5. List of the 74,499 SNPs on chromosome 5, filtered based on homozygous genotypes and segregation ratio

Table S6. Significantly associated SNPs on chromosome 12 and 16 based on 10kb-window FST analysis

Table S7. List of the 2,190 bin markers (bin length, number of SNPs, and genotype), related to Figure 4

Table S8. Construction of the genetic linkage map using the RIL population, related to Figures 5 and 6

Table S9. Detailed information on the 118 protein-coding genes in the BgSRR1, related to Table 1

Table S10. List of significantly enriched GO terms within the BgSRR1 region, related to Figures 7A to 7F
