# Supplementary material for: Genomic basis of schistosome resistance in a molluscan vector of human schistosomiasis
Source: iScience. 2024 Dec 2;28(1):111520. doi: 10.1016/j.isci.2024.111520 (PMC11699755; doi:10.1016/j.isci.2024.111520)
Supplement: Document S1. Figure S1 [file mmc1.pdf]

iScience, Volume *28*

Supplemental information

Genomic basis of schistosome resistance in a molluscan vector of human schistosomiasis

Si-Ming Zhang, Guiyun Yan, Abdelmalek Lekired, and Daibin Zhong

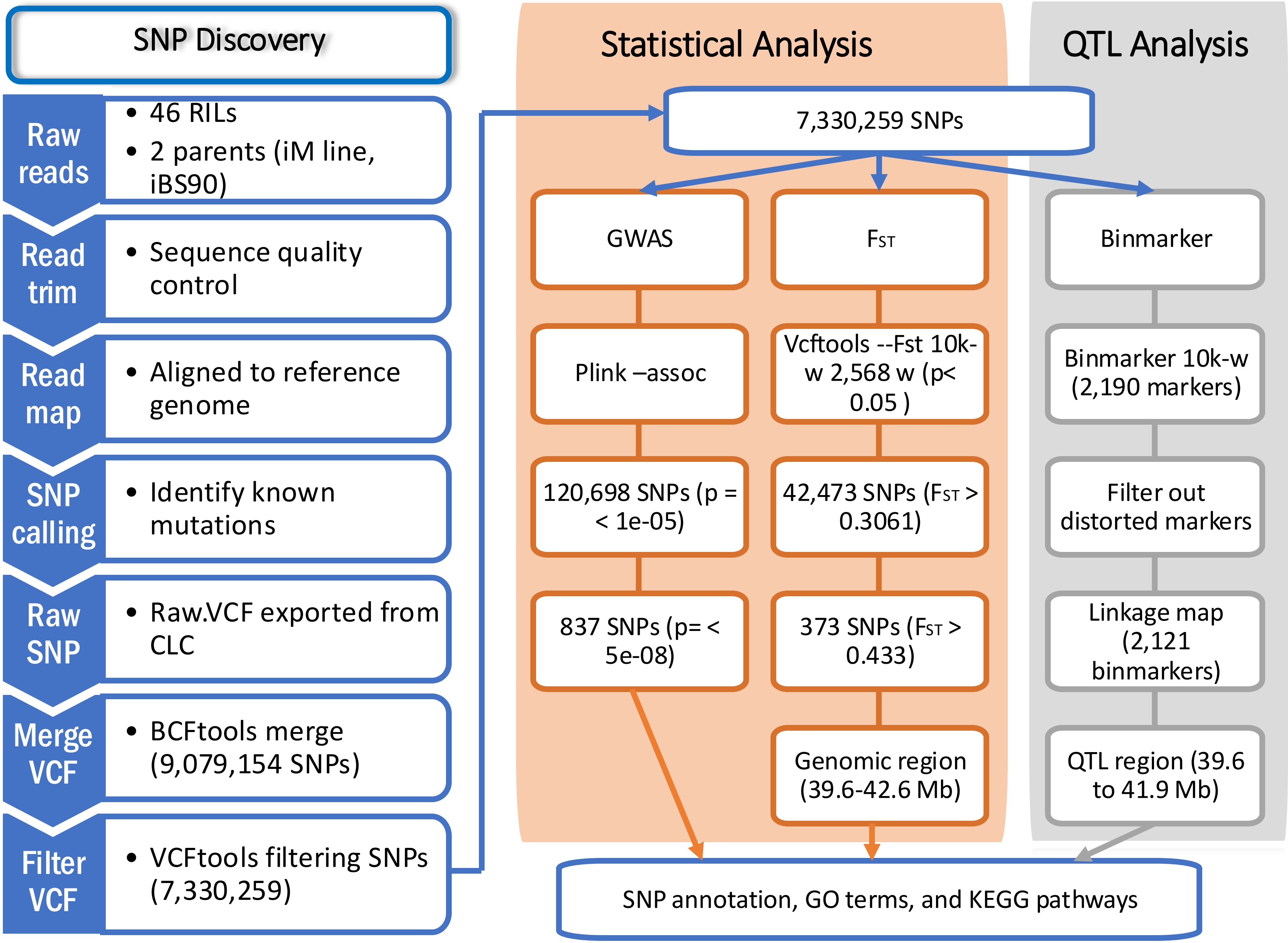

Figure S1. A flowchart of bioinformatic and genetic analyses
